# Performance of *p*-Toluenesulfonic Acid–Based Deep Eutectic Solvent in Denitrogenation: Computational Screening and Experimental Validation

**DOI:** 10.3390/molecules25215093

**Published:** 2020-11-03

**Authors:** Ainul F. Kamarudin, Hanee F. Hizaddin, Lahssen El-blidi, Emad Ali, Mohd A. Hashim, Mohamed K. Hadj-Kali

**Affiliations:** 1 University of Malaya Centre for Ionic Liquids (UMCiL), University of Malaya, Kuala Lumpur 50603, Malaysia; ainulfitree@gmail.com (A.F.K.); alihashim@um.edu.my (M.A.H.); 2 Department of Chemical Engineering, Faculty of Engineering, University of Malaya, Kuala Lumpur 50603, Malaysia; 3 Chemical Engineering Department, King Saud University, P.O. Box 800, Riyadh 11421, Saudi Arabia; lelblidi@KSU.EDU.SA (L.E.-b.); amkamal@ksu.edu.sa (E.A.)

**Keywords:** liquid–liquid extraction, heptane, quinoline, *p*-toluenesulfonic acid, H-NMR

## Abstract

Deep eutectic solvents (DESs) are green solvents developed as an alternative to conventional organic solvents and ionic liquids to extract nitrogen compounds from fuel oil. DESs based on *p*-toluenesulfonic acid (PTSA) are a new solvent class still under investigation for extraction/separation. This study investigated a new DES formed from a combination of tetrabutylphosphonium bromide (TBPBr) and PTSA at a 1:1 molar ratio. Two sets of ternary liquid–liquid equilibrium experiments were performed with different feed concentrations of nitrogen compounds ranging up to 20 mol% in gasoline and diesel model fuel oils. More than 99% of quinoline was extracted from heptane and pentadecane using the DES, leaving the minutest amount of the contaminant. Selectivity was up to 11,000 for the heptane system and up to 24,000 for the pentadecane system at room temperature. The raffinate phase’s proton nuclear magnetic resonance (^1^H-NMR) spectroscopy and GC analysis identified a significantly small amount of quinoline. The selectivity toward quinoline was significantly high at low solute concentrations. The root-mean-square deviation between experimental data and the non-random two-liquid (NRTL) model was 1.12% and 0.31% with heptane and pentadecane, respectively. The results showed that the TBPBr/PTSADES is considerably efficient in eliminating nitrogen compounds from fuel oil.

## 1. Introduction

Nitrogen and sulfur compounds are harmful to the environment and the health of living organisms, including human and aquatic lives. One of the major sources of these compounds is exhaust gases from vehicles. Gasoline and diesel contain nitrogen and sulfur compounds that are not completely extracted during production. The nitrogen content of fuel oil is a precursor to NO*_x_* particle emission into the environment. Most NO*_x_* emissions are anthropogenic, and the US Environmental Protection Agency (EPA) has recommended only 0.2 g/HP-h of these emissions since 2010 [1]. Although emissions have decreased by up to 40%, the EPA estimates that heavy trucks will be responsible for one-third of emissions in 2025.

Conventionally, hydroprocessing units are used in refineries to extract nitrogen and sulfur compounds from fuel oil. However, nitrogen compounds are also detrimental to hydroprocessing units, as they inhibit the efficiency of desulfurization by preventing the essential catalytic reaction in eliminating sulfur compounds [2]. In addition, basic nitrogen compounds, such as quinoline, strongly inhibit hydrodesulfurization through catalyst poisoning and competitive adsorption. Even a small amount of basic nitrogen compounds can lead to fuel oil having >10 ppm of sulfur content [2,3]. They are also a precursor for coke formation in hydroprocessing units [4]. Since sulfur compounds are not completely extracted during desulfurization because of a competitive reaction with nitrogen, it is difficult to adhere to the maximum allowable sulfur concentration set by regulations [5].

The extraction of nitrogen compounds can increase the selectivity for sulfur extraction from fuel oil by 60% [6,7]. Liquid–liquid extraction (LLE) is a convenient, green, and economical method of extracting nitrogen compounds compared to hydro-denitrogenation, which is expensive and requires high hydrogen pressure, a high-temperature unit, and expensive catalysts. In addition, because of the rate-limiting thermodynamic step, an increase in the temperature or hydrogen partial pressure does not support reduction of nitrogen-based polycyclic aromatic hydrocarbons (PAHs) [3]. As a result of the small amount of nitrogen and sulfur present in gasoline, LLE is recommended to extract contaminants in lower-range concentrations.

LLE is widely used to decrease the amount of nitrogen compounds at room temperature and atmospheric pressure. Various organic solvents such as methanol, ethanol, and *N*-methylformamide are used to extract nitrogen compounds from n-hexadecane [8,9,10]. Most of the organic solvents are efficient in extraction processes [11]. However, their physical properties, such as high volatility, low thermal and chemical stability, and toxicity hinder their potential to be widely used on an industrial scale [12,13,14]. In the past few years, ionic liquids (ILs) have been widely used in denitrogenation.

Green deep eutectic solvents (DESs) have been developed as an alternative to conventional organic solvents and ILs. DESs have unique characteristics, such as non-flammability, non-volatility, low vapor pressure, low melting point, and high chemical and thermal stability, which is similar to ILs but with simpler synthesis processes and cheaper raw materials [15]. Ali et al. (2016) showed that DESs made up of a mixture of choline chloride (ChCl) salt and a hydrogen bond donor (HBD) of phenylacetic acid or phenylpropionic acid at a 1:2 molar ratio can efficiently extract both pyridine and carbazole [16]. Both DESs, ChCl:phenylacetic acid and ChCl:phenylpropionic acid, can extract pyridine with an extraction efficiency of 99.2% and 96.3%, respectively, and they can extract carbazole with an extraction efficiency of 98.2% and 97.9%, respectively [17]. Hizaddin et al. (2015) reported extractive denitrogenation from diesel model fuel oil using ILs and DESs. Although ILs showed higher selectivity compared to DESs, their distribution coefficient was low, resulting in a higher volume needed in a single extraction. In addition, phosphonium-based DESs are more efficient in extracting nitrogen compounds compared to ammonium-based DESs [12,17].

Most of the DESs are not as efficient as ILs in extracting basic nitrogen compounds. The IL 1-butyl-3-methylimidazolium bromide with zinc chloride has a high extraction efficiency of 94.95% in extracting basic nitrogen compounds from diesel fuel oil [18]. Separately, Naik et al. (2017) showed that a selectivity of up to 5831 of a DES toward quinoline can be achieved by using the DES methyltriphenylphosphonium bromide and ethylene glycol at a 1:4 molar ratio. The same DES also shows a high selectivity of 3606 toward indoline, which is a non-basic nitrogen compound. These findings are in agreement with Hizaddin et al., who reported that phosphonium-based DESs can be potential solvents for extracting nitrogen compounds [17].

This study developed a green, low-cost DES comprising tetrabutylphosphonium bromide (TBPBr) and *p*-toluenesulfonic acid (PTSA) and investigated for the first time its performance related to the extraction of nitrogen compounds from fuel oil. The aim was to search for the best solvent to extract nitrogen compounds from fuel oil at a low solute concentration, because most of the studies have used a high solute concentration for extracting nitrogen compounds (>10 wt % of nitrogen compounds), while in practice, crude oil contains <10 wt % of nitrogen compounds. The methods were separated into two parts: computational method and experimental validation. The computational method involved quantum chemical calculation using TmoleX software and COSMO-RS calculation using COSMOthermX19 software. However, this was followed by experimental validation, which involved DES synthesis, the preparation of a mixture, extraction experiments, and compositional analysis by GC and proton nuclear magnetic resonance (^1^H-NMR) spectroscopy. We also reported the physical properties of the TBPBr:PTSA 1:1 DES.

## 2. Computational Screening

COnductor-like Screening Model for Real Solvents or COSMO-RS is a statistical thermodynamic property prediction model based on quantum chemical calculations to determine the chemical potential (*µ*) in liquids. COSMO-RS is a two-step approach that includes a COSMO calculation of molecules using density functional theory and statistical thermodynamics of the molecular interactions. In COSMO-RS, the molecules are placed in a conductor as the reference state; then, the charge induced at the surface is calculated and termed as the screening charge as well as stored in a *.cosmo file*. The electrostatic misfit energy (*E*_misfit_), hydrogen bond interaction (*E*_hb_), and van der Waals interaction (*E*_vdW_) represent the molecular interactions in COSMO-RS [19,20]. The surface of the molecules in the liquid can be divided into several segments, and they have their own surface charge density for specific segments. A probability function or known as σ-profile can be generated by applying a local averaging algorithm on the surface charge densities over effective contact segments. The σ-profile is very useful, since it can help to understand the properties and the solvation of the compounds and their mixtures in terms of charge interaction [20].

COSMO-RS is used in this work to predict liquid–liquid equilibrium properties and liquid extraction capability for DES-containing systems. Qualitative screening of ILs using the *σ*-profile and *σ*-potential is useful to determine molecular interactions of compounds and their mixtures. The *σ*-profile is a probability distribution function that shows the amount of surface having a screening charge density *σ*. Negative partial charges of atoms cause a positive screening charge density and vice versa. Predicting the activity coefficient at infinite dilution ($\gamma^{\infty }$) for solutes in a potential solvent using COSMO-RS is a reliable method to screen the solvents quantitatively. $\gamma^{\infty }$ indicate the molecular interaction between the solute and the solvent at a miniscule solute concentration. The predicted $\gamma^{\infty }$ values are used to estimate the selectivity ($S^{\infty }$), capacity ($C^{\infty }$), and performance index ($PI^{\infty }$) at infinite dilution [19]. A total of 59 DESs are screened using COSMO-RS (Table 1).

**Figure 1:**
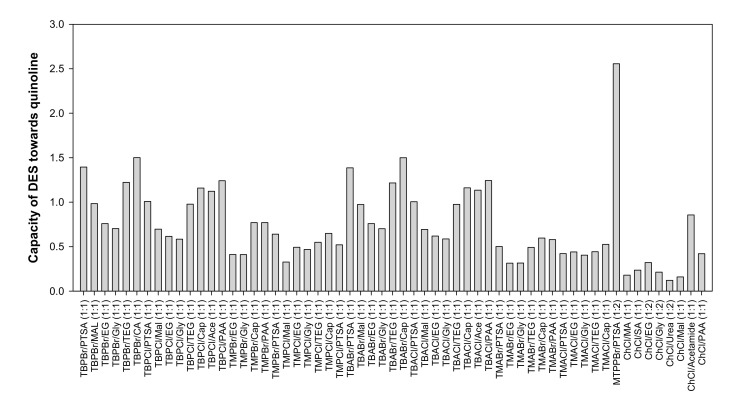
Capacity of quinoline in all DESs.

**Figure 2:**
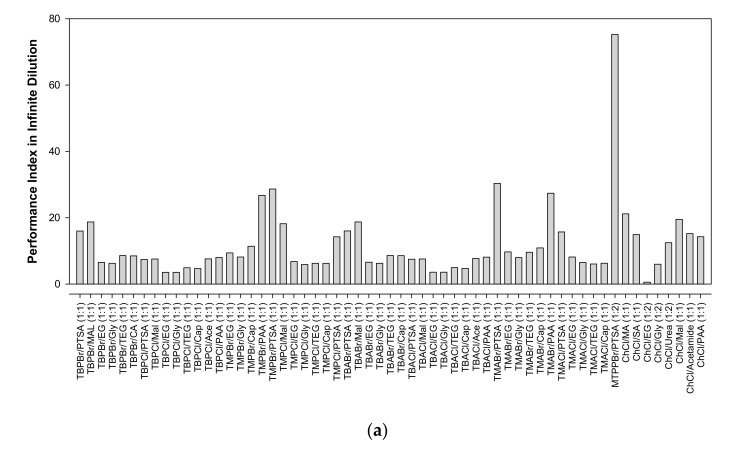

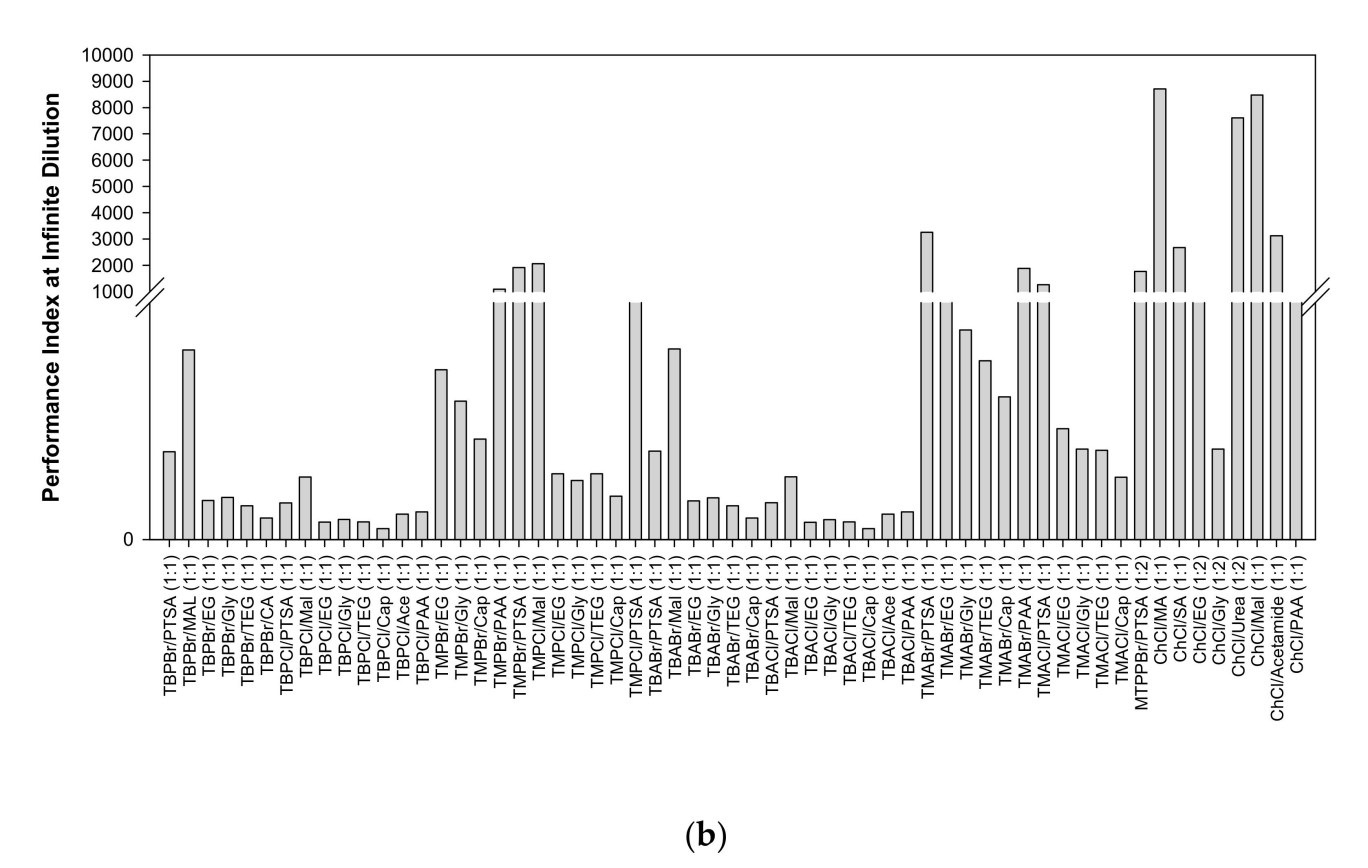
(**a**) Performance index of DES in heptane system, (**b**) Performance index of DES in pentadecane system.

The screening results are illustrated in Figure 1 for their capacity toward quinoline, while Figure 2 shows their performance index. As shown in Figure 2, the highest performance index was for the ChCl-based DES. However, this type of DES has the lowest capacity, which means multiple extraction stages are needed, increasing the cost of extraction for the oil and gas industry. Phosphonium- and ammonium-based DESs do not have a significant difference in selectivity and capacity.

Tetramethyl-based DESs have a relatively high-performance index due to their high selectivity. Their high selectivity depends on the small non-polar chain with weak van der Waals forces interacting with the long hydrocarbon chain from heptane and pentadecane. However, these DESs have low capacity compared to tetrabutyl-based DESs. Since this study focused on extracting a low concentration of basic nitrogen compounds, it was better to select a solvent that has relatively high selectivity and high capacity. High capacity would ensure that all the contaminant was extracted in a single extraction and less solvent was needed. This selection would save on solvent and installation costs, since a small unit would be needed. The screening results showed that a tetrabutyl-based DES with malonic acid as an HBD will be the best solvent for this work, since it has a high-performance index with high selectivity and capacity. However, the DES recrystallizes at room temperature. Hence, we selected DES 1, which comprised TBPBr and PTSA, for this study because of its high capacity and relatively high selectivity in heptane system. In addition, the mixture of TBPBr and PTSA at a molar ratio of 1:1 has not been investigated yet, neither for physical properties nor experimental validation.

**Figure 3:**
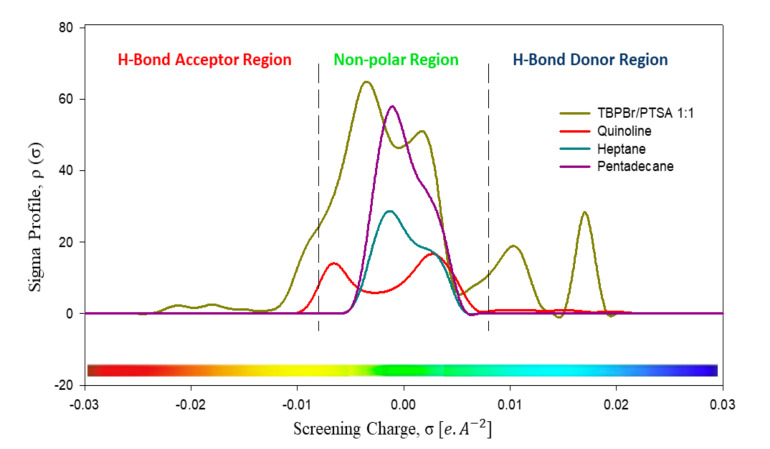
Sigma profiles of all species in the heptane system and in the pentadecane system. The region between −0.008 eA^−2^ < σ < +0.008 eA^−2^ indicates the London dispersion force in heptane. Peaks at σhb > +0.008eA^−2^ indicate hydrogen bonding energy and the presence of hydrogen bond donor, while peaks at σhb < +0.008eA^−2^ indicate the presence of a hydrogen bond acceptor group.

We generated the *σ*-profiles and *σ*-potentials of the selected DES toward model fuel oil. Negative *σ* values indicated positive polarities of the component, while positive *σ* values indicated negative polarities [20]. Figure 3 shows the *σ*-profile of all species. As shown, the heptane and pentadecane peaks were within the range of −0.008 eA^−2^ < *σ* < +0.008 eA^−2^, indicating that they consist of van der Waals forces, which specifically can be London dispersion forces. These forces allow non-polar molecules to have attractive forces and hold together. In both systems, quinoline showed almost a symmetric *σ*-profile with a peak resulting from polarized hydrogen and the *π*-face of aromatic rings [20]. The nitrogen atom in quinoline is responsible for hydrogen bond interaction energy and acts as a hydrogen bond acceptor. This hydrogen bond acceptor group participates in hydrogen bond interaction with the hydrogen atom from heptane and DES [21]. The peak at +0.017 eA^−2^ for the DES represents the bromide atom in the species, while the peak at 0.011 eA^−2^ represents the oxygen atoms in PTSA. This peak showed that the DES compound was able to have hydrogen bonding interaction with quinoline and act as hydrogen bond donor. This peak showed that the DES compound was able to have a hydrogen bonding interaction with quinoline and act as a hydrogen bond donor. The largest peak for the DES represents the four butyl chains around the phosphonium atom, causing the cation to become less polar. The non-polar interaction between this DES and hydrocarbon showed that a small amount of hydrocarbon will be extracted along with quinoline during a liquid–liquid extraction process.

**Figure 4:**
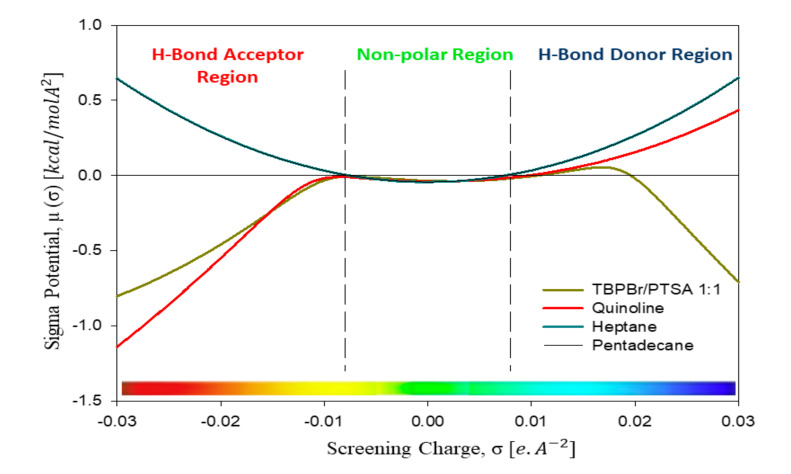
Sigma potential of all species in the heptane system and the pentadecane system. The region between −0.008 eA^−2^ < σ < +0.008 eA^−2^ indicates the London dispersion force in heptane. Peaks at σhb > +0.008eA^−2^ indicate hydrogen bonding energy and the presence of a hydrogen bond donor, while peaks at σhb < +0.008eA^−2^ indicate the presence of a hydrogen bond acceptor group.

Figure 4 shows the *σ*-potential, which indicates the affinity of a component in a mixture toward other compounds. A higher negative value of *µ*(*σ*) indicates an increasing interaction between molecules, while a higher positive value indicates an increase in repulsive behavior. The horizontal axis refers to the region for the hydrogen bond donor and hydrogen bond acceptor. Hydrocarbons have a parabolic potential curve, and interaction can only occur at the non-polar region. The potential curves of heptane and pentadecane are similar in the non-polar region. Therefore, they have the same affinity toward DES. The hydrogen bonding ability of this DES is apparently an advantage, since quinoline and the DES show a possible interaction between them. As shown, the *σ*-profile of the nitrogen-based aromatic compound was slightly complementary with the DES.

However, some of the heptane and pentadecane was extracted because of interaction in the non-polar region. Aliphatic has its significantly small electrostatic field (almost 0) because of the saturated carbon–hydrogen and carbon–carbon bonds [22]. Therefore, the DES can extract some aliphatic along with aromatic compounds. The extraction of nitrogen compounds is high because of the hydrogen bonding between the nitrogen atom of quinoline and the –OH group of the DES. In addition, hydrogen bonding also occurs between the oxygen atoms of the DES and any hydrogen in quinoline.

## 3. Experimental Validation

### 3.1. Materials and Methods

We purchased heptane, pentadecane, quinoline, *p*-toluenesulfonic acid monohydrate, tetrabutylphosphonium bromide, and deuterated chloroform from Merck, Germany. All chemicals were used without further purification. Table 2 lists the source, purity, and CAS number for all chemicals used in this work.

### 3.2. DES Synthesis and Characterization

TBPBr and monohydrate-PTSA have been weighed on an electrical weighing balance according to the molar ratio 1:1 of salt to HBD. The DES was synthesized using a dried round-bottom flask (50 mL) to avoid any contamination with water. Known quantities of salt and PTSA were added to the flask. The mixtures were stirred on a heating plate at a rotational speed of 400 rpm at 353 K until a homogenous and stable yellow DES solution was formed. For example, to prepare about 20 g of TBABr/PTSA DES with the molar ratio 1 to 1, 13.74 g of TBABr and 7.60 g of PTSA were used. The mixtures were left for a week to make sure that the solution is stable.

We measured the density and viscosity as a function of temperature using the HPDT Density Meter (Mettler Toledo, Columbus, OH, USA) and the Rotational Viscometer Rheolab QC (Anton Paar, Graz, Austria). We also determined the melting point (*T*_m_) or glass transition temperature (*T*_g_) using a DSC; Mettler Toledo). The sample was cooled from 313 to 203 K at a cooling rate of 1 K/min for two cycles under nitrogen gas. The decomposition temperature was determined using thermogravimetric analysis. In addition, ^1^H-NMR and Fourier transform infrared (FTIR) analysis were used to confirm the structure and purity of DES.

### 3.3. Liquid–Liquid Extraction Protocol

We formed the model fuel oil by mixing the different amounts (4, 8, 10, 12, 16, and 20 wt %) of quinoline in alkane to vary the feed composition for the tie line generation. Next, the TBPBr:PTSA 1:1 DES and model fuel oil were mixed in close-capped vials at a 1 g to 1 g mass ratio such that a heterogenous mixture was formed. The vials with different solute compositions were sealed with PARAFILM tape to prevent any component loss. Next, the vials were agitated using an incubator shaker at a shaking speed of 200 rpm for 3 h at room temperature and then kept for another 4 h to let the mixtures settle until two distinctly separated phases were formed. Each experiment was triplicated, and the uncertainty was calculated and reported from molar compositions. Finally, the top and bottom layers of each sample were collected and sent for gas chromatography (GC) analysis, and the absence of the DESs in the top layers was confirmed by ^1^H-NMR spectroscopy.

### 3.4. GC and ^1^H-NMR Spectroscopy Analysis

Trace GC-2010 (Shimadzu Corporation, Kyoto, Japan) equipment to analyze the liquid phases at equilibrium comprised a flame ionization detector and an HP-5 column (5% diphenyl 95% dimethylpolysiloxane, 30 m, 0.32 mm, 0.25 µm). Helium with split mode was used as a carrier gas. Table 3 lists the optimum conditions for GC analysis for the quinoline/alkane system. For GC LLE analysis, a sample of 10 µL from rafinate phase (hydrocarbon-rich phase) and 100 µL from the extract phase (DES-rich phase) were taken by a microliter syringe and diluted in 1 mL of acetonitrile. Then, 1 µL of each sample was injected into the GC column. However, calibration curves were first constructed by plotting the area ratio of quinoline/n-alkanes peaks against the specified molar ratio in the range of quinoline concentration investigated in this study. As can be seen in Figure S1 (provided as Supplementary Materials), both ratios are proportional, and excellent agreement was obtained.

We performed ^1^H-NMR spectroscopy for each pure TBPBr/PTSA and raffinate phases by dissolving one drop of selected compound in 0.5 mL of deuterated chloroform. The solution was analyzed by HNMR spectrometer Bruker Ultrashield Plus (400 MHz).

## 4. Results and Discussion

### 4.1. DES Physical Properties

**Figure 5:**
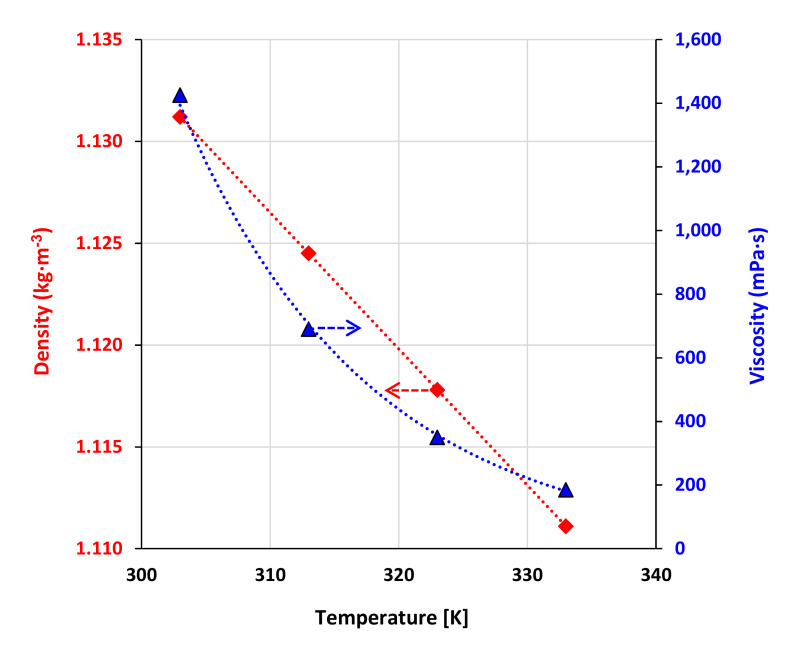
Density and viscosity as a function of temperature. The density and viscosity decrease as temperature increases. Red line represents the density and blue line represents the viscosity of the DES. The arrows show the y-axis of respective fitted line.

The TBPBr:PTSA DES with a 1:1 molar ratio formed a yellow liquid upon heating and remained a liquid at room temperature after 24 h. We measured the density of both DESs in a temperature range of 308–333 K and 1 atm. Figure 5 illustrates the values, and Table S1 shows the data. The linear trend of density with temperature was expressed as follows:

(1) $$\rho \;\left(\frac{g}{cm^{3}}\right)=a+bT\left(K\right)$$

where ρ is the density in *g/cm*^3^, *T* is the temperature in K, and *a* and *b* are adjustable parameters. Table S2 summarizes the parameters. The higher molar ratio of the DES resulted in higher density (Figure 5). The TBPBr:PTSA 1:1 DES had denser properties compared to the TBPCl:PTSA 1:1 DES because of the denser anion type used. This result is in agreement with the literature, indicating that the salt type affects the DES’s volumetric properties [23,24]. Higher HBD concentration in the DES increases the free molar volume, and therefore, the density increases.

The viscosity of both DESs was measured in a temperature range of 308–333 K and 1 atm. Figure 5 illustrates the values, and Table S1 shows the data. The viscosity was expressed as a function of temperature using the Arrhenius equation:

(2) $$µ=Ae^{B/RT}$$

where *µ* is the dynamic viscosity, *A* is the pre-exponential constant, *B* is the activation energy, *R* is the gas constant, and *T* is the temperature in Kelvin. Table S3 shows the adjustable fitting parameters.

The TBPBr:PTSA 1:1 DES is more viscous, and the values are almost doubled because of the presence of the bromide anion compared to the TBPCl:PTSA 1:1 DES. However, a decrease in viscosity with a decrease in the salt concentration is consistent with previous work. The electrostatic interactions of the anion get weaker as the salt concentration decreases. Therefore, viscosity decreases [23].

### 4.2. Melting Point and Decomposition Temperature

**Figure 6:**
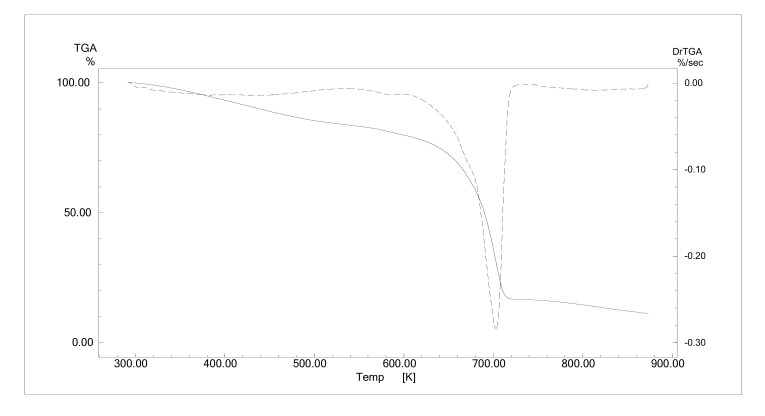
Dynamic TGA analysis of tetrabutylphosphonium bromide (TBPBr):*p*-toluenesulfonic acid (PTSA) (1:1). The solid line illustrates the reduction of DES mass, while the dashed line illustrates the degradation peak.

A differential scanning calorimeter (DSC) was used to determine *T*_m_ or *T*_g_. However, we did not observe any melting point peak in both DESs. Instead, we observed a glass transition from DSC measurement (Figure S2). The *T*_g_ of the TBPBr:PTSA 1:1 DES, 235 K, was slightly lower compared to the TBPCl:PTSA DES. The bromide ion weakens intermolecular interactions, lowering *T*_g_. The TBPBr:PTSA 1:1 DES is expected to have the same decomposition temperature range as the TBPCl:PTSA 1:1 DES, which is 680 K (Figure 6). The type of anion of the salt does not differ much since they are from the same halogen group. The graph in Figure 6 shows 20% mass loss at 373 K because of evaporation of water, followed by 65% mass reduction because of DES decomposition.

### 4.3. Spectroscopic Studies

**Figure 7:**
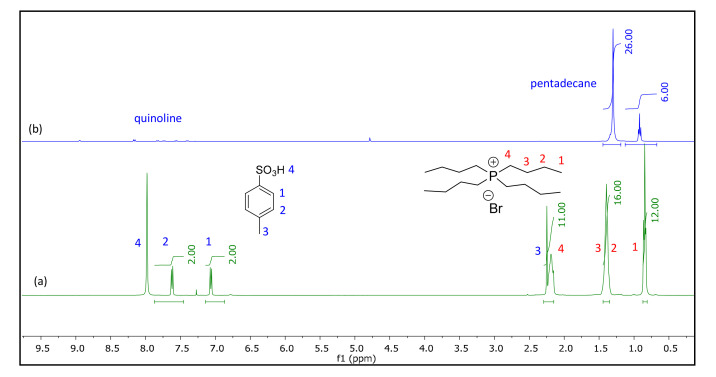
NMR spectra of TBPBr/PTSA DES (1:1, molar ratio) in CDCl3.

We used ^1^H-NMR and FTIR spectroscopy to analyze the behavior of the TBPBr:PTSA 1:1 DESs. The FTIR spectra of the formed DES can be referred to in the Supporting Material (Figure S3) both with different signals detected for 1H-NMR for TBPBr:PTSA (1:1) DES (Table S8). In the ^1^H-NMR spectra (Figure 7), the identification peaks for quinoline were between 7.4 and 8.9 ppm. The peak at 8.9 ppm was the representative for the hydrogen atom of quinoline. The identification peaks for the TBPBr:PTSA 1:1 DES were taken at hydrogen atoms at 2.28 ppm for the TBP cation and 7.7 ppm for PTSA. Finally, n-heptane showed peaks at 0.88 ppm, which represented the methyl group, and 1.3 ppm, which represented the –CH_2_ group.

^1^H-NMR spectra showed that the peak of the salt (TBPBr) and HBD (PTSA) are the same as the pure compound with integration for 1:1 molar ratio, indicating that the salt and HBD do not react with each other. Instead, their intermolecular interaction causes the mixture to be a stable DES, as shown by the FTIR spectra. The peaks in the region of 2800–1800 cm^−1^ which represent the symmetric and asymmetric stretching of SO_3_, disappeared after DES formation, indicating that hydrogen bonding occurred between PTSA and TBPBr instead of between PTSA and H_2_O (Figure 7).

### 4.4. Experimental Results

Table 4 and Table 5 show the experimental results for the TBPBr/PTSA (1:1) (1) + quinoline (2) + heptane/n-pentadecane (3) system, along with the capacity (distribution coefficient, *D*) of the TBPBr/PTSA 1:1 DES and its selectivity, *S*, toward nitrogen-based PAHs. For both systems and at all feed compositions in model fuel oil, the fraction of alkane in the extract phase was almost equal to 0.999. The DES extracted >99% of the quinoline from heptane and pentadecane, leaving the minutest amount of the contaminant. Therefore, both LLE systems produced >99% pure model fuel oil using the TBPBr/PTSA 1:1 DES. The reduction of hydrocarbons from each model fuel oil in the experimental process confirmed the simulation of *σ*-profiles of all these components in COSMO-RS. The non-polar interaction force between these two species causes the hydrocarbons to be slightly extracted along with quinoline. The raffinate phase showed that no DES component was extracted in the hydrocarbon-rich phase.

In most cases, an increase in the aromatic compound concentration decreases selectivity [20], which is consistent with our results. However, in this study, the distribution coefficient and selectivity were significantly higher compared to screening results. The distribution coefficient is defined as the ratio of the concentration of a certain component in the extract phase to its concentration in the raffinate phase. Selectivity is defined as the distribution coefficient ratio of the capacity of the targeted component to that of the model fuel oil. The mathematical simplification is as follows:

(3) $$D=\frac{x_{2}^{\prime }}{x_{2}^{\prime \prime }}$$

(4) $$S=\frac{D_{2}}{D_{3}}=\frac{x_{2}^{\prime }x_{3}^{\prime \prime }}{x_{2}^{\prime \prime }x_{3}^{\prime }}$$

where *x*_2_ is the solute concentration in the model fuel oil and *x*_3_ is the concentration of the model fuel oil. Superscripts ′ and ″ refer to extract and raffinate phases, respectively.

The high distribution coefficient and selectivity were probably why the DES is suitable for extracting nitrogen-based polycyclic compounds. The distribution coefficient of quinoline is significantly high, and it is very important for industry to decrease the amount of solvent needed for large-scale LLE.

The distribution coefficient for all systems was >1, indicating that a large amount of quinoline can be extracted in one extraction cycle. Increasing the quinoline mole fraction in the raffinate phase, along with distribution coefficient, showed that this extraction process is viable for low and high feed concentrations of quinoline. In addition, a small amount of DES is required for multistage extraction, since the DES can extract almost all the quinoline in one extraction cycle. The selectivity of all systems was also >1, indicating that extraction is feasible. In addition, the TBPBr/PTSA equilibrium concentration in the raffinate phase was 0 with no cross-contamination, and it has been clearly shown in Figure 7. These findings can be crucial for industry, as the TBPBr/PTSA 1:1 DES can replace common industrial solvents that require unnecessary purification, resulting in solvent loss. Additionally, the TBPBr/PTSA 1:1 DES only requires a small operation unit because of the fewer extraction cycles required.

### 4.5. Consistency Test

We used Bachman [25], Hand [26], and Othmer –Tobias [27] equations to check the reliability of ternary LLE (Liquid-liquid equilibria) tie lines using Equations (5)–(7), respectively:

(5) $$x_{2}^{\prime }=a\left(\frac{x_{2}^{\prime }}{x_{3}^{\prime \prime }}\right)+b$$

(6) $$ln\frac{x_{2}^{\prime \prime }}{x_{3}^{\prime \prime }}=c\left(ln\frac{x_{3}^{\prime \prime }}{x_{1}^{\prime }}\right)+d$$

(7) $$ln\frac{\left(1-x_{3}^{\prime \prime }\right)}{x_{3}^{\prime \prime }}=e\left(ln\frac{1-x_{1}^{\prime \prime }}{x_{1}^{\prime }}\right)+f.$$

In Equation (5), the Bachman equation, $x_{2}^{\prime }$ and $x_{3}^{\prime \prime }$ represent the mole fraction of quinoline in the extract phase and that of heptane or pentadecane in the raffinate phase, respectively, and *a* and *b* are fitting parameters. In Equation (6), the Hand equation, $x_{2}^{\prime \prime },\;x_{3}^{\prime \prime },$ and $x_{1}^{\prime }$ represent the mass fraction of quinoline in the raffinate phase, the mole fraction of fuel oil in the raffinate phase, and the mass fraction of the TBPBr:PTSA 1:1 DES in the extract phase, respectively, and *c* and *d* are fitting parameters. All mole fractions were obtained from GC analysis, and the fitting process was done by Excel. Both correlation tests were aligned to each other. Table 6 lists the parameters of Bachman, Hand, and Othmer–Tobias correlations.

### 4.6. Extraction Efficiency

Clean fuel oil production depends on the efficiency of quinoline extraction from the model fuel oil (Table 7). Since the TBPBr/PTSA 1:1 DES extracted >99% of quinoline, the production of 100% nitrogen-free fuel oil is feasible using this DES. A single extraction cycle can ensure >99% quinoline extraction up to 20 wt % quinoline for the heptane and pentadecane system. The negligible quinoline concentration in the separation product does not affect the extraction of sulfur compounds in the subsequent stage. Table 7 shows the efficiency of quinoline extraction from 4–20 wt % quinoline.

### 4.7. Comparison between COSMO-RS Predictions and Experimental Measurements

**Figure 8:**
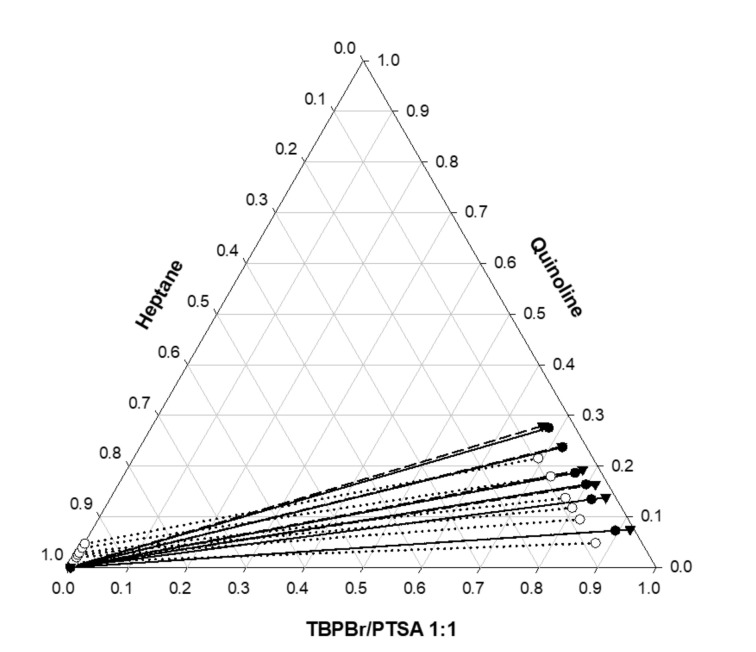
Ternary phase diagram for TBPBr/PTSA + quinoline + heptane at 25 °C. The solid line represents experimental data, dotted line represents COSMO-RS prediction, and the dashed line represents non-random two-liquids (NRTL) LLE prediction.

**Figure 9:**
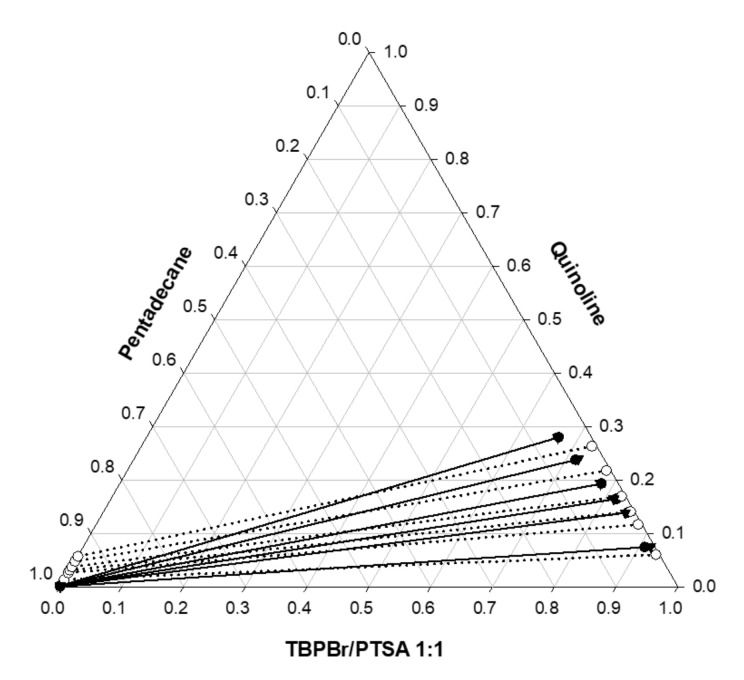
Ternary phase diagram for TBPBr/PTSA + quinoline + pentadecane at 25 °C. The solid line represents experimental data, dotted line represents COSMO-RS prediction, and the dashed line represents NRTL LLE prediction.

We used root-mean-square deviation (RMSD) to compare the values calculated by LLE from COSMO-RS (Tables S4 and S5) and experimental measurements at each tie line with the following equation:

(8) $$\mathrm{RMSD}\;\left(\%\right)=100\sqrt{\overset{m}{\underset{k=1}{\sum }}\overset{c}{\underset{i=1}{\sum }}\overset{2}{\underset{j=1}{\sum }}\frac{{\left(x_{ik}^{j,exp}-x_{ik}^{j,cal}\right)}^{2}}{2mc}}$$

where *m* is the number of tie lines, *c* is the number of components, and *j* is the number of phases. Therefore, $x_{ik}^{j,exp}$ is the concentration of component *i* in the *j* phase at the *k* tie line. The heptane system had an RMSD of 3.55%, while the pentadecane system has an RMSD of 3.43%, indicating that both calculated and experimental compositions were consistent with each other. Figure 8 and Figure 9 illustrate their deviations.

### 4.8. NRTL LLE Modeling

To find phase compositions in LLE, the isothermal liquid–liquid flash needs to be calculated and solved. The following equations are part of the flash:

Material balance:

(9) $$x_{i}-\left(1-\omega \right)x_{i}^{L1}-\omega x_{i}^{L2}=0,i=1,N_{c}$$

Equilibrium:

(10) $$x_{i}^{L1}\gamma_{i}^{L1}-x_{i}^{L2}\gamma_{i}^{L2}=0,\;i=1,N_{c}$$

Equation of summation:

(11) $$\sum_{i}x_{i}^{L1}-\sum_{i}x_{i}^{L2}=0.$$

Here, ω is the liquid–liquid splitting ratio; $x_{i}$ is the composition of component *i* in the mixture; $x_{i}^{L1}$ is the composition of component *i* in the liquid phase *L*1; $x_{i}^{L2}$ is the composition of component *i* in the liquid phase *L*2; $\gamma_{i}^{L1}$ and $\gamma_{i}^{L2}$ are the activity coefficients of component *i* in liquid phases 1 and 2, respectively; and $N_{c}$ is the number of constituents.

We used non-random two-liquid (NRTL) to estimate the activity coefficient as follows:

(12) $$ln\gamma_{i}=\frac{\sum_{j}\tau_{ji}G_{ji}x_{j}}{\sum_{j}G_{ji}x_{j}}+\underset{k}{\sum }\frac{G_{ji}x_{j}}{\sum_{k}G_{kj}x_{k}}\left(\tau_{ij}-\frac{\sum_{k}\tau_{kj}G_{kji}x_{k}}{\sum_{k}G_{kj}x_{k}}\right)$$

where $lnG_{ij}=-\alpha_{ij}\tau_{ij}$; $\alpha_{ij}=\alpha_{ji}$; $\tau_{ij}=\frac{g_{ij}-g_{ii}}{RT}=\frac{C_{ij}}{RT}$; and $\tau_{ii}=\tau_{jj}=0$. Model development was achieved within the Simulis^®^ Thermodynamics environment.

To estimate the binary interaction parameters *C_ij_* and *C_ji_*, the RMSD between calculated and experimental solubilities of each component in each phase is minimized, as described by LLE flash equations. In addition, $\alpha_{ij}$ is the non-randomness parameter; when $\alpha_{ij}$ = 0, the mixture is completely random. Renonet al. (1968) reported that the non-randomness parameter normally lies between 6 and 12, so $\alpha_{ij}$ = 0.1–0.3 [28]. In this study, we fixed the value for all binary combinations at 0.2 on the basis of our previous successful fitting of the NRTL model for ternary LLE data for DES-containing systems.

Table 8 lists the values of the NRTL binary interaction parameters regressed in this study for each binary. To conserve coherence between this and previous studies on ILs and DESs, we have initialized the binary interaction parameters between pentadecane and quinoline based on our previous studies about quinoline and hexadecane without any modifications [17]. Similarly, we kept the binary interaction parameters between quinoline and the TBPBr:PTSA DES constant in both ternary systems despite a change in the hydrocarbon compound. The RMSD between the NRTL model and experimental data was 1.12% and 0.31% with heptane and pentadecane, respectively, indicating that NRTL correlation represents experimental data very well. This excellent fitting is also obvious from ternary diagrams and the NRTL composition can be referred to Tables S6 and S7.

### 4.9. Comparison between This and Other Studies

Table 9 and Table 10 summarize the ternary LLE data collected from the literature at 298.15 K and atmospheric pressure. Pyridine is a common solute used in LLE with heptane as the model fuel oil compound representative of gasoline. Choline-based DESs show the best selectivity because there is no pyridine in the extract phase. However, the distribution is low (0.17–1.16) compared to others (>2). Naik et al. (2017) showed that quinoline extraction can be done with methyltriphenylphosphonium bromide and ethylene glycol in a 1:4 ratio, resulting in a high distribution coefficient and selectivity [3]. Warrag et al. (2020) obtained a high distribution coefficient and selectivity while extracting pyridine from n-heptane solution using the same DES [29]. These results indicate that this DES can extract basic and non-basic nitrogen-based PAHs. However, its low distribution coefficient might lead to multistage extraction. Our TBPBr/PTSA 1:1 DES has a better distribution coefficient compared to others in one extraction cycle, and so it can cut the cost of multistage equipment installation and the amount of extracting solvent required.

Hizaddin et al. (2015, 2016) extracted nitrogen compounds from hexadecane as a diesel representative. However, the selected ILs and DESs were effective toward non-basic nitrogen compounds, pyrrole and indoline. Basic nitrogen compounds, quinoline and pyridine, have a lower distribution coefficient and selectivity compared to non-basic nitrogen compounds probably because of delocalized electrons in pyridine and quinolone, decreasing the strain and increasing their stability compared to pyrrole, a 5-membered ring. Compared to our TBPBr/PTSA 1:1 DES, TBAB/EG (1:2) and TBPB/EG (1:2) DESs have higher capability to extract quinoline because of their high selectivity. However, our TBPBr/PTSA 1:1DES can extract double the amount of quinoline in a single extraction cycle (distribution coefficient = 5–16) compared to TBAB/EG and TBPB/EG DESs (distribution coefficient = 3–7).

## 5. Conclusions

We synthesized a neoteric DES for use in denitrogenation of fuel oil by LLE. Since the HBD plays a vital role in extracting nitrogen compounds, a high-acidity HBD is required to create enough affinity toward basic nitrogen compounds. However, the acidity should also be appropriate in order to avoid unnecessary chemical reactions. The TBPBr/PTSADES at a 1:1 molar ratio extracted >99% of the nitrogen-based aromatic hydrocarbon with selectivity up to 11,000 for the heptane system and 99% with selectivity up to 24,000 for the pentadecane system at room temperature. The TBPBr/PTSA 1:1 DES has better intermolecular interaction toward quinoline because of its high polarity level compared to hydrocarbons. In addition, this DES is recommended for the separation of low-nitrogen-concentration fuel oil, and it is the finishing step before the product is sent to the desulfurization unit. The effect of the solvent-to-feed ratio on extraction efficiency can be continually investigated. A low ratio will save more cost on the amount of solvent required in addition to preventing more hydrocarbon loss during extraction.

## Figures and Tables

**Table 1:** List of deep eutectic solvents (DESs) used in COnductor like Screening Model for Real Solvents (COSMO-RS) screening.

No	Salt/HBA	HBD	Abbreviation
1	Tetrabutylphosphonium bromide	*p*-Toluenesulfonic acid	TBPBr/PTSA (1:1)
2	Tetrabutylphosphonium bromide	Malonic acid	TBPBr/MAL (1:1)
3	Tetrabutylphosphonium bromide	Ethylene glycol	TBPBr/EG (1:1)
4	Tetrabutylphosphonium bromide	Glycerol	TBPBr/Gly (1:1)
5	Tetrabutylphosphonium bromide	Tetraethylene glycol	TBPBr/TEG (1:1)
6	Tetrabutylphosphonium bromide	Caproic acid	TBPBr/CA (1:1)
7	Tetrabutylphosphonium chloride	*p*-Toluenesulfonic acid	TBPCl/PTSA (1:1)
8	Tetrabutylphosphonium chloride	Malonic acid	TBPCl/Mal (1:1)
9	Tetrabutylphosphonium chloride	Ethylene glycol	TBPCl/EG (1:1)
10	Tetrabutylphosphonium chloride	Glycerol	TBPCl/Gly (1:1)
11	Tetrabutylphosphonium chloride	Tetraethylene glycol	TBPCl/TEG (1:1)
12	Tetrabutylphosphonium chloride	Caproic acid	TBPCl/Cap (1:1)
13	Tetrabutylphosphonium chloride	Acetic acid	TBPCl/Ace (1:1)
14	Tetrabutylphosphonium chloride	Phenylacetic acid	TBPCl/PAA (1:1)
15	Tetrametyhlphosphonium bromide	Ethylene glycol	TMPBr/EG (1:1)
16	Tetrametyhlphosphonium bromide	Glycerol	TMPBr/Gly (1:1)
17	Tetrametyhlphosphonium bromide	Caproic acid	TMPBr/Cap (1:1)
18	Tetrametyhlphosphonium bromide	Phenylacetic acid	TMPBr/PAA (1:1)
19	Tetrametyhlphosphonium bromide	*p*-Toluenesulfonic acid	TMPBr/PTSA (1:1)
20	Tetrametyhlphosphonium chloride	Malonic acid	TMPCl/Mal (1:1)
21	Tetrametyhlphosphonium chloride	Ethylene glycol	TMPCl/EG (1:1)
22	Tetrametyhlphosphonium chloride	Glycerol	TMPCl/Gly (1:1)
23	Tetrametyhlphosphonium chloride	Tetraethylene glycol	TMPCl/TEG (1:1)
24	Tetrametyhlphosphonium chloride	Caproic acid	TMPCl/Cap (1:1)
25	Tetrametyhlphosphonium chloride	*p*-Toluenesulfonic acid	TMPCl/PTSA (1:1)
26	Tetrabutylammonium bromide	*p*-Toluenesulfonic acid	TBABr/PTSA (1:1)
27	Tetrabutylammonium bromide	Malonic acid	TBABr/Mal (1:1)
28	Tetrabutylammonium bromide	Ethylene glycol	TBABr/EG (1:1)
29	Tetrabutylammonium bromide	Glycerol	TBABr/Gly (1:1)
30	Tetrabutylammonium bromide	Tetraethylene glycol	TBABr/TEG (1:1)
31	Tetrabutylammonium bromide	Caproic acid	TBABr/Cap (1:1)
32	Tetrabutylammonium chloride	*p*-toluenesulfonic acid	TBACl/PTSA (1:1)
33	Tetrabutylammonium chloride	Malonic acid	TBACl/Mal (1:1)
34	Tetrabutylammonium chloride	Ethylene Glycol	TBACl/EG (1:1)
35	Tetrabutylammonium chloride	Glycerol	TBACl/Gly (1:1)
36	Tetrabutylammonium chloride	Tetraethylene glycol	TBACl/TEG (1:1)
37	Tetrabutylammonium chloride	Caproic acid	TBACl/Cap (1:1)
38	Tetrabutylammonium chloride	Acetic acid	TBACl/Ace (1:1)
39	Tetrabutylammonium chloride	Phenylacetic acid	TBACl/PAA (1:1)
40	Tetramethylammonium bromide	*p*-Toluenesulfonic acid	TMABr/PTSA (1:1)
41	Tetramethylammonium bromide	Ethylene glycol	TMABr/EG (1:1)
42	Tetramethylammonium bromide	Glycerol	TMABr/Gly (1:1)
43	Tetramethylammonium bromide	Tetraethylene glycol	TMABr/TEG (1:1)
44	Tetramethylammonium bromide	Caproic acid	TMABr/Cap (1:1)
45	Tetramethylammonium bromide	Phenylacetic acid	TMABr/PAA (1:1)
46	Tetramethylammonium chloride	*p*-Toluenesulfonic acid	TMACl/PTSA (1:1)
47	Tetramethylammonium chloride	Ethylene Glycol	TMACl/EG (1:1)
48	Tetramethylammonium chloride	Glycerol	TMACl/Gly (1:1)
49	Tetramethylammonium chloride	Tetraethylene glycol	TMACl/TEG (1:1)
50	Tetramethylammonium chloride	Caproic acid	TMACl/Cap (1:1)
* 51	Methyltriphenylphosphonium bromide	*p*-Toluenesulfonic acid	MTPPBr/PTSA (1:2)
52	Choline chloride	Malic acid	ChCl/MA (1:1)
53	Choline chloride	Succinic acid	ChCl/SA (1:1)
* 54	Choline chloride	Ethylene glycol	ChCl/EG (1:2)
* 55	Choline chloride	Glycerol	ChCl/Gly (1:2)
* 56	Choline chloride	Urea	ChCl/Urea (1:2)
57	Choline chloride	Malonic acid	ChCl/Mal (1:1)
* 58	Choline chloride	Acetamide	ChCl/Acetamide (1:2)
59	Choline chloride	Phenylacetic acid	ChCl/PAA (1:1)

* The molar ratio for this DES is (1:2).

**Table 2:** List of purchased chemicals used in this project.

No.	Compound Name	CAS No.	Purity
1	n-Heptane	142-82-5	≥0.99
2	Pentadecane	629-62-9	≥0.99
3	Quinoline	91-22-5	≥0.99
4	Tetrabutylphosphonium bromide	3115-68-2	>0.98
5	*p*-Toluenesulfonic acid monohydrate	6192-52-5	>0.98
6	Deuterated chloroform (*CDCl_3_*)	865-49-6	≥0.998

**Table 3:** Gas chromatography operating conditions.

Parameter	
Temperature of injector (K)	558.15
Temperature of detector (K)	558.15
Carrier gas pressure (Kpa)	60
Oven program	333.15 K for 6 min
	333.15 K to 513.15 K
	Rate: 20 K/min

**Table 4:** Compositional analysis for systems containing quinoline, heptane, and DES TBPBr/PTSA (1:1) at room temperature along with distribution ratio, *D*, and selectivity, *S*.

DES-Rich Phase			Hydrocarbon-Rich Phase			*D*	*S*
$x_{1}^{\prime }$	$x_{2}^{\prime }$	$x_{3}^{\prime }$	$x_{1}^{\prime \prime }$	$x_{2}^{\prime \prime }$	$x_{3}^{\prime \prime }$		
***TBPBr/PTSA* (1:1) (1) *+ quinoline* (2) *+ heptane* (3)**							
0.8940	0.0726	0.0334	0	0.0002	0.9998	363	10,866
0.8223	0.1343	0.0434	0	0.0003	0.9997	448	10,312
0.7982	0.1638	0.0380	0	0.0004	0.9996	410	10,772
0.7683	0.1866	0.0451	0	0.0004	0.9996	467	10,340
0.7213	0.2372	0.0415	0	0.0006	0.9994	395	9520
0.6790	0.2755	0.0455	0	0.0007	0.9993	394	8644

**Table 5:** Compositional analysis for systems containing quinoline, pentadecane, and DES TBPBr/PTSA (1:1) at room temperature along with distribution ratio, *D*, and selectivity, *S*.

DES-Rich Phase			Hydrocarbon-Rich Phase			*D*	*S*
$x_{1}^{\prime }$	$x_{2}^{\prime }$	$x_{3}^{\prime }$	$x_{1}^{\prime \prime }$	$x_{2}^{\prime \prime }$	$x_{3}^{\prime \prime }$		
***TBPBr/PTSA* (1:1) (1) *+ quinoline* (2) *+ pentadecane* (3)**							
0.9095	0.0738	0.0167	0	0.0003	0.9997	246	14,726
0.8499	0.1387	0.0114	0	0.0005	0.9995	277	24,321
0.8182	0.1642	0.0176	0	0.0006	0.9994	274	15,540
0.7797	0.1928	0.0275	0	0.0008	0.9992	241	8,757
0.7157	0.2372	0.0471	0	0.0011	0.9989	216	4,573
0.6672	0.2795	0.0533	0	0.0014	0.9986	200	3,740

**Table 6:** Parameters of Bachman and Hand correlation for each ternary LLE system and the values of regression coefficient.

Ternary System	Bachman			Hand			Othmer –Tobias		
	*a*	*b*	*R* ^2^	*c*	*d*	*R^2^*	*e*	*f*	*R* ^2^
TBPBr/PTSA + Quinoline + heptane	0.999	0.000	1.000	0.790	−5.803	0.972	0.896	−5.732	0.970
TBPBr/PTSA + Quinoline + pentadecane	0.998	0.0002	1.000	0.955	−5.701	0.984	0.955	−5.932	0.999

**Table 7:** Summary of quinoline’s extraction efficiency at different concentration in heptane and pentadecane.

wt % of Quinoline in Feed	Mole Fraction of Quinoline in Heptane		Extraction Efficiency	Mole Fraction of Quinoline in Pentadecane		Extraction Efficiency
	Feed	Product		Feed	Product	
4	0.0313	0.0002	99.4%	0.06413	0.0003	99.5%
8	0.0632	0.0003	99.5%	0.12512	0.0005	99.6%
10	0.0794	0.0004	99.5%	0.15450	0.0006	99.6%
12	0.0957	0.0004	99.6%	0.18318	0.0008	99.6%
16	0.1288	0.0006	99.5%	0.23854	0.0011	99.5%
20	0.1625	0.0007	99.6%	0.29136	0.0014	99.5%

**Table 8:** The values of NRTL binary interaction parameters.

*i*–*j*	$\tau_{ij}$	$\tau_{ji}$
Quinoline–heptane	−172.1	251.9
Quinoline–pentadecane	−160.3	229.0
*n*-heptane–TBPBr/PTSA (1:1)	4180.6	1588.2
pentadecane–TBPBr/PTSA (1:1)	4111.2	644.0
Quinoline–TBPBr/PTSA (1:1)	4264.7	1596.6

**Table 9:** Experimental data on the use of ionic liquids (ILs) and DESs for extractive denitrogenation from heptane and pentadecane.

DES/IL	Model Fuel Oil	Solute	Distribution Coefficient	Selectivity	Extraction Efficiency	Ref.
TBPBr/PTSA (1:1)	n-heptane	quinoline	467–363	10,800–8600	≥99%	This work
TBPBr/PTSA (1:1)	n-pentadecane	quinoline	246–200	14,700–3700	≥99%	This work
MTPPBr/Gly (1:4)	n-hexane	pyridine	2.677–1.589	839.5–26.1	NA	[29]
MTPPBr/EG (1:4)	n-heptane	pyridine	2.644–1.396	1268–91.7	NA	[29]
MTPPBr/EG (1:4)	n-heptane	quinoline	29.33–15.58	5831–1149	NA	[3]
MTPPBr/EG (1:4)	n-heptane	quinoline	12.40–9.48	2398–1347	NA	
[HiQuin][SCN]	n-heptane	pyridine	6.33–1.27	269–4.65	NA	[30]
[C8iQuin][SCN]	n-heptane	pyridine	5.70–1.10	104–1.67	NA	
[HiQuin][NTf(2)]	n-heptane	pyridine	10.2–1.55	102–4.41	NA	
[Oquin][NTf2]	n-heptane	pyridine	9.30–1.47	73.00–3.42	NA	
[C2mim][EtSO_4_]	n-heptane	pyridine	NA	NA	27.52–70.58%	[31]
[C5mim][Tf2N]	n-heptane	pyridine	NA	NA	45.62–78.59%	
[C6mmPy][Tf2N]	n-heptane	pyridine	NA	NA	39.21–75.69%	
[bzmim][Tf2N]	n-heptane	pyridine	NA	NA	36.19–70.17%	
[C7mmim][Tf2N]	n-heptane	pyridine	NA	NA	32.16–70.64%	
[C10mmim][Tf2N]	n-heptane	pyridine	NA	NA	32.48–77.11%	
[EMIM][SCN]	n-heptane	pyridine	3.85–1.12	1208.90–6.80	NA	[32]
[DMIM][MP]	n-heptane	pyridine	1.19–0.61	49.6–6.3	NA	
Bet/PPG (1:4)	n-heptane	pyridine	3.46–1.90	No Heptane in Extract Phase	NA	[33]
Bet/PPG (1:5)	n-heptane	pyridine	3.24–1.89	No Heptane in Extract Phase	NA	
ChCl/Gly (1:2)	n-hexane	pyridine	0.89–1.16	No Hexane in Extract Phase	51%	[34]
ChCl/Urea (1:2)	n-hexane	pyridine	0.17–1.03	No Hexane in Extract Phase	NA	
[BMIM][TCM]	n-heptane	pyridine	11.30–2.47	540.3–30.3	NA	[35]
[BMMOR][TCM]	n-heptane	pyridine	9.20–1.38	609.3–5.90	NA	
[BMPY][TCM]	n-heptane	pyridine	9.42–1.46	578.8–10.7	NA	
[C4mim]Br/ZnCl_2_	n-hexadecane	Basic N	NA	NA	94.95%	[18]
TBABr/ EG (1:2)	n-hexadecane	pyridine	4.22–2.93	1,228–418	NA	[17]
		quinoline	5.00–3.56	4,955–3,229	NA	
TBPBr/ EG (1:2)	n-hexadecane	pyridine	4.60–3.24	437–158	NA	
		quinoline	7.80–3.71	594–141	NA	
[EMIM][EtSO_4_]	n-hexadecane	pyridine	3.17	1,023	NA	[12]
		quinoline	2.33	353	NA	
[EMIM][MeSO_3_]	n-hexadecane	pyridine	3.26	573	NA	
		quinoline	4.35	491	NA	
[EMPY][EtSO_4_]	n-hexadecane	pyridine	6.46	1800	NA	
		quinoline	4.17	685	NA	
TEAC	Model wash oil	quinoline	3.2	NA	NA	[36]
TEAC	Model wash oil	quinoline	1.3	NA	NA	[36]
TEMAC	Model wash oil	quinoline	2.0	NA	NA	[36]

**Table 10:** List of full names of ILs and DESs and their abbreviation used in Table 9.

Name of DESs/ILs	Abbrev.
Methyltriphenylphosphonium bromide/Ethylene Glycol (1:4)	MTPPBr/EG (1:4)
Methyltriphenylphosphonium bromide/Ethylene Glycol (1:4)	MTPPBr/EG (1:4)
Methyltriphenylphosphonium bromide/Ethylene Glycol (1:4)	MTPPBr/EG (1:4)
Methyltriphenylphosphonium bromide/Ethylene Glycol (1:4)	MTPPBr/EG (1:4)
N-Hexylisoquinolinium thiocyanate	[HiQuin][SCN]
N-Octylisoquinolinium thiocyanate	[C8iQuin][SCN]
N-Hexylisoquinoliniumbis{(trifluoromethyl) sulfonyl} imide	[HiQuin][NTf(2)]
N-Octylquinoliniumbis{(trifluoromethyl) sulfonyl}imide	[Oquin][NTf2]
1-Ethyl-3-methylimidazolium ethyl sulfate	[C2mim][EtSO4]
1-Pentyl-3-methylimidazolium bis(trifluoromethylsulfonyl)imide	[C5mim][Tf2N]
1-Hexyl-3,5-dimethylpyridinium bis(trifluoromethylsulfonyl)imide	[C6mmPy][Tf2N]
1-Benzyl-3-methylimidazolium bis(trifluoromethylsulfonyl)imide	[bzmim][Tf2N]
1-Heptyl-2,3-dimethylimidazolium bis(trifluoromethylsulfonyl)imide	[C7mmim][Tf2N]
1-Decyl-2,3-dimethylimidazolium bis(trifluoromethylsulfonyl)imide	[C10mmim][Tf2N]
1-ethyl-3-methylimidazolium thiocyanate	[EMIM][SCN]
1,3-Dimethylimidazolium methylphosphonate	[DMIM][MP]
Betaine/Propylene Glycol (1:4)	Bet/PPG (1:4)
Betaine/Propylene Glycol (1:5)	Bet/PPG (1:5)
1-Butyl-3-methylimidazolium tricyanomethanide	[BMIM][TCM]
1-Butyl-1-methylmorpholinium(4-butyl-4-methyl-morpholinium) tricyanomethanide	[BMMOR][TCM]
1-Butyl-4-methylpyridinium tricyanomethanide	[BMPY][TCM]
1-butyl-3-methylimidazolium bromide/zinc chloride	[C4mim]Br/ZnCl2
1-Ethyl-3-methylimidazolium ethyl sulfate	[EMIM][EtSO4]
1-Ethyl-3-methylimidazolium methanesulfonate	[EMIM][MeSO3]
1-Ethyl-3-methylpyridinium ethyl sulfate	[EMPY][EtSO4]
Tetraethylammonium chloride	TEAC
Tetraethyl-methylammonium chloride	TEMAC

## Supplementary Materials

Table S1: Density and viscosity of DES TBPBr:PTSA (1:1), Table S2: Fitting parameters for linear fit of the density as function of temperature, Table S3: Fitting parameters for viscosity as function of the temperature according to Arrhenius equation, Table S4: Prediction values of LLE of quinoline from n-heptane by COSMO-RS, Table S5: Prediction values of LLE of quinoline from pentadecane by COSMO-RS, Table S6: Prediction values of LLE in extraction of quinoline from heptane by NRTL, Table S7: Prediction values of LLE in extraction of quinoline from pentadecane by NRTL, Table S8: Different signal detected for 1H-NMR for TBPBr:PTSA (1:1) DES, Table S9: Different signal in raffinate phase of pentadecane system detected by 1H-NMR, Figure S1: Calibration curve of heptane and pentadecane for GC, Figure S1: Glass transition temperature of TBPBr/PTSA (1:1). The solid line illustrates the heat flow of DES, Figure S2: FTIR analysis for DES TBPBr:PTSA (1:1) where blue indicates the tetrabutylphosphonium salt, green indicates *p*-toluenesulfonic acid and red indicates the DES.
