# Supplementary material for: Performance of p-Toluenesulfonic Acid–Based Deep Eutectic Solvent in Denitrogenation: Computational Screening and Experimental Validation
Source: Molecules. 2020 Nov 3;25(21):5093. doi: 10.3390/molecules25215093 (PMC7662348; doi:10.3390/molecules25215093)
Supplement: Supplementary file 1 [file molecules-25-05093-s001.pdf]

molecules

Supporting Material for Publication

Performance of *p*-Toluenesulfonic Acid–Based Deep Eutectic Solvent in Denitrogenation: Computational Screening and Experimental Validation

Ainul F. Kamarudin[1,2], Hanee F. Hizaddin[1,2*], Lahssen El-blidi[3], Emad Ali[3], Mohd A. Hashim[1,2] and Mohamed K. Hadj-Kali[3*]

[1]University of Malaya Centre for Ionic Liquids (UMCiL), University of Malaya, 50603 Kuala Lumpur, Malaysia.
[2]Department of Chemical Engineering, Faculty of Engineering, University of Malaya, 50603 Kuala Lumpur, Malaysia.
[3] Chemical Engineering Department, King Saud University, P.O. Box 800, Riyadh 11421, Saudi Arabia.
*Corresponding authors: hanee@um.edu.my; mhadjkali@ksu.edu.sa

List of tables

Table S1: Density and viscosity of DES TBPBr:PTSA (1:1).
Table S2: Fitting parameters for linear fit of the density as function of temperature.
Table S3: Fitting parameters for viscosity as function of the temperature according to Arrhenius equation.
Table S4: Prediction values of LLE of quinoline from n-heptane by COSMO-RS
Table S5: Prediction values of LLE of quinoline from pentadecane by COSMO-RS.
Table S6: Prediction values of LLE in extraction of quinoline from heptane by NRTL.
Table S7: Prediction values of LLE in extraction of quinoline from pentadecane by NRTL.
Table S8: Different signal detected for ^{1}H-NMR for TBPBr:PTSA (1:1) DES.
Table S9: Different signal in raffinate phase of pentadecane system detected by ^{1}H-NMR.

List of figures

Figure S1: Calibration curve of heptane and pentadecane for GC.
Figure S1: Glass transition temperature of TBPBr/PTSA (1:1). The solid line illustrates the heat flow of DES.
Figure S2: FTIR analysis for DES TBPBr:PTSA (1:1) where blue indicates the tetrabutyl-phosphonium salt, green indicates p-toluenesulfonic acid and red indicates the DES.

molecules MDPI

Table S1: Density and viscosity of DES TBPBr:PTSA (1:1).

Temperature (°C)	Density ($^{g}/_{cm^3}$)	Viscosity (mPa.s)
	TBPBr:PTSA (1:1)	TBPBr:PTSA (1:1)
30	1.1312	1,425
40	1.1245	690
50	1.1178	350
60	1.1111	185

Table S2: Fitting parameters for linear fit of the density as function of temperature.

DES	a	b	R^2
TBPBr:PTSA (1:1)	-0.0007	1.1515	0.9999

Table S3: Fitting parameters for viscosity as function of the temperature according to Arrhenius equation.

DES	A	B	R^2
TBPBr:PTSA (1:1)	2.00E-07	57,151	0.9964

Table S4: Prediction values of LLE of quinoline from n-heptane by COSMO-RS.

DES-rich phase			Hydrocarbon-rich phase			*D*	*S*
x'_1	x'_2	x'_3	x''_1	x''_2	x''_3		
TBPBr/PTSA (1:1) (1) + quinoline (2) + n-heptane (3)							
0.872	0.048	0.079	0.000	0.010	0.990	4.854	60.47
0.822	0.095	0.083	0.001	0.020	0.980	4.794	56.71
0.798	0.117	0.085	0.001	0.025	0.975	4.762	54.87
0.777	0.137	0.086	0.001	0.029	0.970	4.733	53.27
0.730	0.180	0.090	0.001	0.038	0.960	4.670	49.81
0.691	0.215	0.094	0.001	0.047	0.952	4.613	46.91

Table S5: Prediction values of LLE of quinoline from pentadecane by COSMO-RS.

DES-rich phase			Hydrocarbon-rich phase			*D*	*S*
x'_1	x'_2	x'_3	x''_1	x''_2	x''_3		
TBPBr/PTSA (1:1) (1) + quinoline (2) + pentadecane (3)							
0.934	0.060	0.006	0.000	0.013	0.987	4.534	757.0
0.878	0.116	0.006	0.000	0.025	0.974	4.571	700.0
0.853	0.140	0.007	0.000	0.031	0.969	4.586	675.9
0.824	0.169	0.007	0.001	0.037	0.963	4.604	647.9
0.775	0.217	0.007	0.001	0.047	0.952	4.633	601.6
0.729	0.263	0.008	0.001	0.056	0.943	4.660	559.0

molecules

MDPI

Table S6: Prediction values of LLE in extraction of quinoline from heptane by NRTL.

DES-rich phase			Hydrocarbon-rich phase			D	S
x'1	*x'2*	*x'3*	*x''1*	*x''2*	*x''3*		
TBPBr/PTSA (1:1) (1) + quinoline (2) + heptane (3)							
0.918	0.075	0.007	0	0.0001	0.9999	885	118,573
0.845	0.138	0.017	0	0.0002	0.9998	731	44,226
0.815	0.164	0.022	0	0.0002	0.9998	670	30,856
0.778	0.193	0.029	0	0.0003	0.9997	600	20,858
0.719	0.239	0.042	0	0.0005	0.9995	488	11,606
0.666	0.279	0.055	0	0.0007	0.9993	393	7,122

Table S7: Prediction values of LLE in extraction of quinoline from pentadecane by NRTL.

DES-rich phase			Hydrocarbon-rich phase			D	S
x'1	*x'2*	*x'3*	*x''1*	*x''2*	*x''3*		
TBPBr/PTSA (1:1) (1) + quinoline (2) + pentadecane (3)							
0.918	0.075	0.007	0.000	0.000	1.000	861	115,476
0.845	0.138	0.016	0.000	0.000	1.000	710	43,151
0.815	0.164	0.022	0.000	0.000	1.000	651	30,118
0.779	0.193	0.029	0.000	0.000	1.000	582	20,365
0.719	0.239	0.042	0.000	0.001	0.999	474	11,335
0.666	0.279	0.055	0.000	0.001	0.999	381	6,958

Table S8: Different signal detected for ^{1}H-NMR for TBPBr:PTSA (1:1) DES.

TBPBr:PTSA (1:1)	Signal	Shift (ppm)	H atom
Tetrabutylphosphonium Bromide	1	0.92	12
	2	1.41	8
	3	1.45	8
	4	2.34	8
p-Toluenesulfonic Acid	1	7.61	2
	2	7.17	2
	3	8.10	3
	4	2.34	1

Table S9: Different signal in raffinate phase of pentadecane system detected by ^{1}H-NMR.

Chemical	Signal	Shift (ppm)	H atom
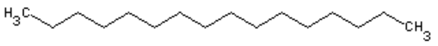	CH_2	1.34	26
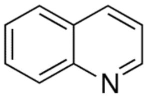	CH	8.96	1

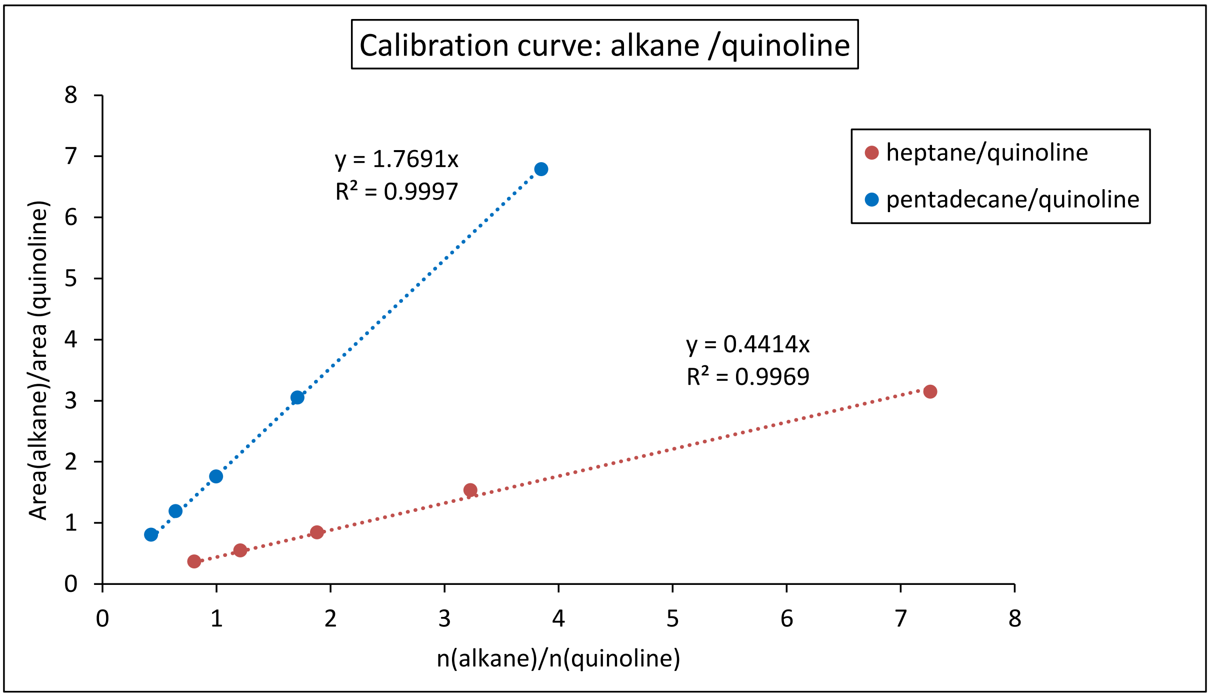

Figure S2: Calibration curve of heptane and pentadecane for GC.

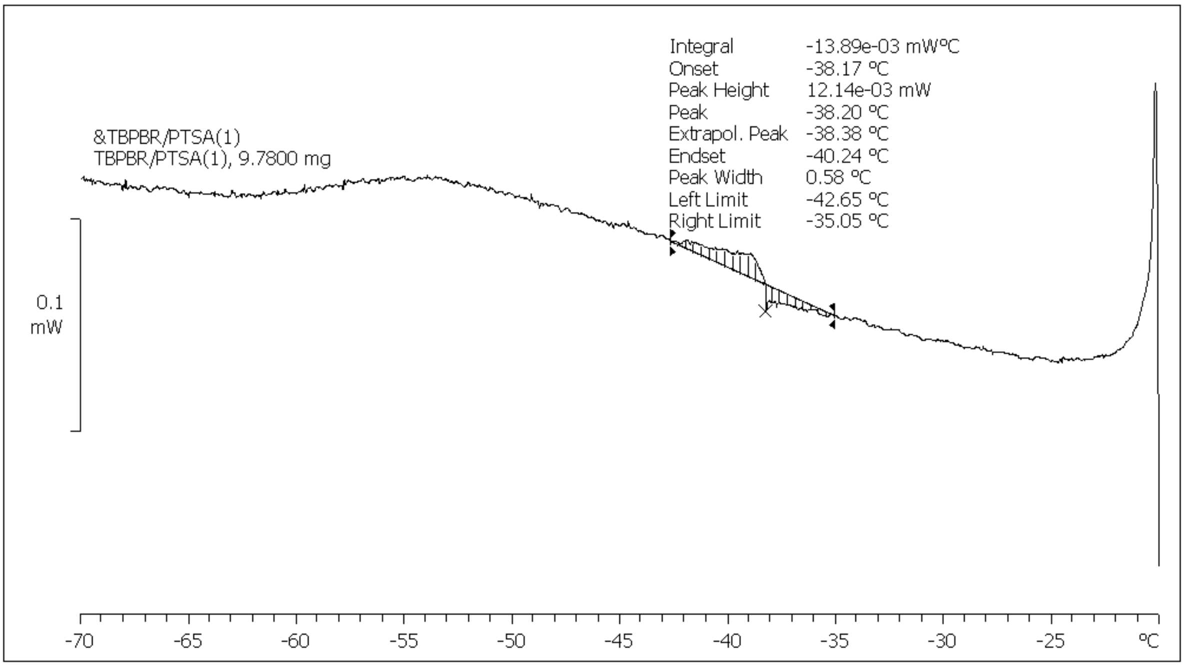

Figure S3: Glass transition temperature of TBPBr/PTSA (1:1). The solid line illustrates the heat flow of DES.

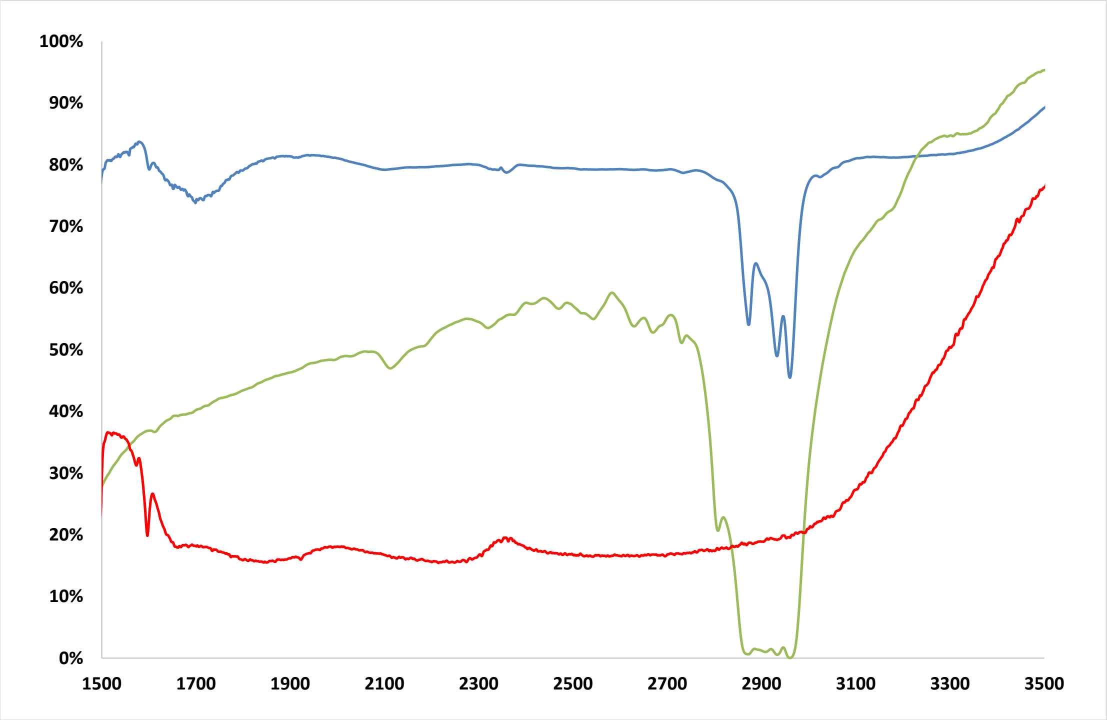

Figure S4: FTIR analysis for DES TBPBr:PTSA (1:1) where blue indicates the tetrabutylphosphonium salt, green indicates p-toluenesulfonic acid and red indicates the DES.
